# Transport of Glial Cell Line-Derived Neurotrophic Factor into Liposomes across the Blood-Brain Barrier: *In Vitro* and *in Vivo* Studies

**DOI:** 10.3390/ijms15033612

**Published:** 2014-02-27

**Authors:** Shaoling Wu, Guoqi Li, Xiao Li, Caina Lin, Ding Yu, Shuo Luan, Chao Ma

**Affiliations:** 1 Rehabilitation Department, Sun Yat-sen Memorial Hospital, Sun Yat-sen University, 107 Yanjiang West Road, Guangzhou 510120, Guangdong, China; E-Mails: 13660183777@126.com (S.W.); lixiao204011987@sina.com (X.L.); lincaina826@126.com (C.L.); 13654456833@163.com (D.Y.); 15018761213@126.com (S.L.); 2 Department of Cardiology, Sun Yat-sen Memorial Hospital, Sun Yat-sen University, Guangzhou 510120, Guangdong, China; E-Mail: liguoqi09@163.com

**Keywords:** glial cell line-derived neurotrophic factor, target sterically stabilized liposomes, blood-brain barrier

## Abstract

Glial cell line-derived neurotrophic factor (GDNF) was encapsulated into liposomes in order to protect it from enzyme degradation *in vivo* and promote its permeability across the blood-brain barrier (BBB). In this study, GDNF conventional liposomes (GDNF-L) and GDNF target sterically stabilized liposomes (GDNF-SSL-T) were prepared. The average size of liposomes was below 90 nm. A primary model of BBB was established and evaluated by transendothelial electrical resistance (TEER) and permeability. This BBB model was employed to study the permeability of GDNF liposomes *in vitro*. The results indicated that the liposomes could enhance transport of GDNF across the BBB and GDNF-SSL-T had achieved the best transport efficacy. The distribution of GDNF liposomes was studied *in vivo*. Free GDNF and GDNF-L were eliminated rapidly in the circulation. GDNF-SSL-T has a prolonged circulation time in the blood and favorable brain delivery. The values of the area under the curve (AUC_(0–1 h)_) in the brain of GDNF-SSL-T was 8.1 times and 6.8 times more than that of free GDNF and GDNF-L, respectively. These results showed that GDNF-SSL-T realized the aim of targeted delivery of therapeutic proteins to central nervous system.

## 1. Introduction

Neurodegenerative diseases represent a major socioeconomic burden and unimaginable misery for millions of sufferers and their families around the world. Glial cell line-derived neurotrophic factor (GDNF), neurotrophin nerve growth factor (NGF) and brain-derived neurotrophic factor (BDNF) are three important trophic factors that regulate neural ontogeny to maintain optimum function [1]. A reduction in level of one or more of these proteins may lead to at least some of the symptoms of Parkinson’s, Alzheimer’s and Huntington’s diseases. Replacement strategies are often considered as potential therapeutics for these neurodegenerative diseases.

GDNF is a distant member of the transforming growth factor β superfamily that was originally isolated from the rat B49 glial cell line [2]. It is expressed throughout the central nervous system (CNS) during development [3], and is mainly expressed and detected in neurons of adult brain [4]. GNDF is considered to be one of the strongest neuroprotectants for dopaminergic neurons. Studies had shown that GDNF is critical for the maintenance and survival of adult dopamine neurons [5] that it can promote survival of mesencephalic dopamine neurons in culture [2], as well as improve survival and re-growth of dopaminergic neurons in adult brain after injury [6,7]. Besides, GDNF is essential in the development and survival of motor neurons, the development of sympathetic and sensory neurons, and in hippocampal synaptogenesis [8,9].

Parkinson’s disease (PD) is one of the major neurodegenerative disorders in middle and old age. This disease arises from a progressive degeneration of dopaminergic neurons in the substantia nigra and is characterized by resting tremor, bradykinesia, rigidity, and postural instability [10]. Many studies, carried out in a wide variety of rodent and primate models of Parkinson’s disease, have demonstrated the efficacy of GDNF [11–13], with clinical trials currently in progress [14]. A number of strategies for GDNF delivery to the brain have been investigated in animal models of PD, including intracerebral injections of recombinant GDNF protein [11], implantation of encapsulated GDNF-secreting cells [15] and intrastriatal delivery of GDNF gene [14,16]. However, all the above approaches are invasive and not suitable for long-term clinical application.

As therapeutic proteins with higher molecular weight, GDNF cannot be transported easily across the blood-brain barrier (BBB) to produce a significant effect following normal administration such as intravascular injection or oral administration. Recently, liposomes have been widely used as delivery vehicles to increase uptake into the brain *in vivo* [17]. To obtain better targeting efficiency, targeted liposomes have been proposed [18].

In this paper, GDNF conventional liposomes (GDNF-L) and GDNF targeted sterically stabilized liposomes (GDNF-SSL-T) were prepared at first, and the permeability of these two GDNF liposomes *in vitro* BBB model and the uptake in brain *in vivo* were then studied.

## 2. Results

### 2.1. Characterization of Liposomes

The average size, encapsulation efficiency (ee%) and recovery efficiency (re%) of GDNF liposomes were shown in Table 1.

### 2.2. Evaluation of the BBB Model

The BBB model was developed by the coculture of rat brain capillary endothelial cells (BCECs) and astrocytes (ACs), and its transendothelial electrical resistance (TEER) and permeability were measured. In our study, TEER value of the coculture BCECs/ACs was 360 ± 35 Ωcm^2^ while the maximum TEER of BCECs alone was only about 40 Ωcm^2^, much lower than that of *in vitro* BBB model. On the contrary, the permeability of Horseradish peroxidase (HRP) showed that it was 3.70% on the coculture BCECs, and only 0.53% on the coculture BCECs/ACs. All the data indicated the primary BBB model had main characterizations of the BBB.

### 2.3. Transport Measurements of GDNF Liposomes on the *in Vitro* BBB Model

**Figure 1.**
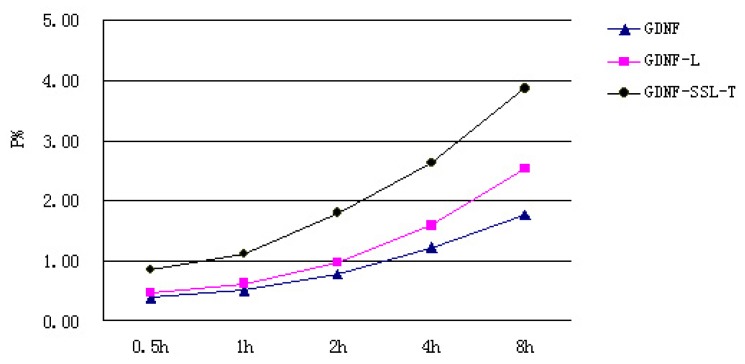
The permeability of GDNF, GDNF-L and GDNF-SSL-T on the BBB model *in vitro n* = 3.

When the BCECs/ACs was confluent, which was judged by TEER primarily, they were employed as *in vitro* BBB model to evaluate the permeability of GDNF liposomes. The permeability of GDNF liposomes was shown in Figure 1. We can see from the chart that the order of permeability on the *in vitro* BBB model was GDNF-SSL-T > GDNF-L > GDNF.

### 2.4. Distribution of GDNF Liposomes in Serum and Brain

**Figure 2.**
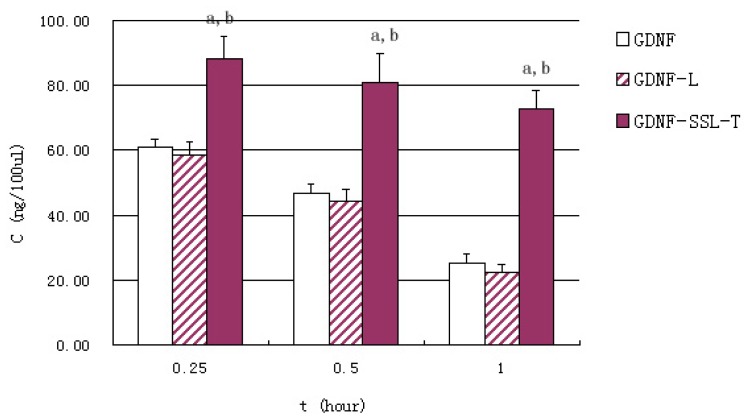
GDNF concentration in serum 0.25, 0.5 and 1 h after intravenous injection of three GDNF liposomes. The results were represented as means ± S.D. (*n* = 6) and compared by Student’s *t*-test. a: *p* < 0.01 *versus* GDNF; b: *p* < 0.01 *versus* GDNF-L.

**Figure 3.**
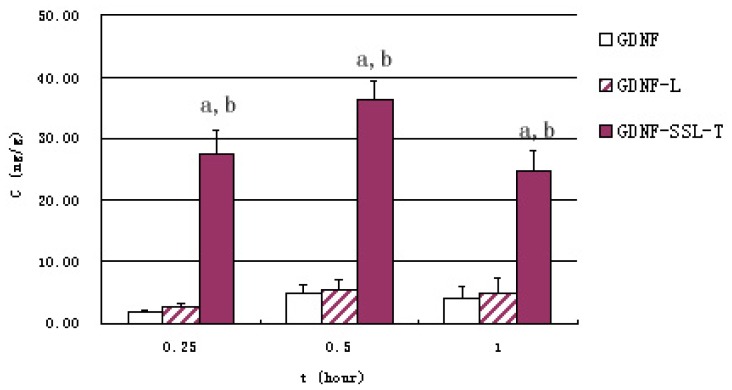
GDNF content in brain 0.25, 0.5 and 1 h after intravenous injection of three GDNF liposomes The results were represented as means ± S.D. (*n* = 6) and compared by Student’s *t*-test. a, *p* < 0.01 *versus* GDNF; b, *p* < 0.01 *versus* GDNF-L.

Figure 2 represents serum GDNF concentration at given intervals after intravenous administration in three groups *in vivo* studies. Free GDNF and GDNF-L were eliminated rapidly in the circulation, while the clearance of GDNF-SSL-T in the blood was significantly slower (*p* < 0.01). The contents of GDNF of each group in the brain at 0.25, 0.5, 1 h were listed in Figure 3. The content of brain GDNF was significantly higher in GDNF-SSL-T group than those of the other two groups at all the three time point (*p* < 0.01). The values of the area under the curve (AUC_(0–1 h)_) in the brain of GDNF, GDNF-L and GDNF-SSL-T groups were 3.30 ± 0.69, 3.94 ± 0.67 and 26.8 ± 2.27 ng/g.h, respectively.

## 3. Discussion

The BBB is a unique structure which is formed by the brain endothelial cells lined with cerebral capillaries, together closely with perivascular astrocytic end-foot, neurons, and pericytes [19]. Tight junction (TJ) between cerebral microvascular endothelial cells is the important structure and functional base of BBB, which allows BBB to maintain a constant and optimal microenvironment for neurons and protects CNS from exogenous toxicants [20]. But on the other hand, this structure also constitutes the most redoubtable obstacle for drug delivery for the treatment of brain disorders. All large-molecule agents and more than 98% of pharmaceutical and small-molecule drugs are blocked by BBB [21]. Besides size, charge, chemical structure and lipophilicity are also main characteristic influencing the permeability through the BBB. In order to facilitate drugs entry to CNS, many noninvasive techniques have been developed. Among these approaches, liposomes seem to be one of the most promising ones. The modified liposomes can enhance drug delivery to the brain with documented advantages, including lipophilic properties, increased drug-loading capacity, biocompatibility, biodegradability, minimal toxicity and versatile structural characteristics that permit easy surface decoration [22].

As strong neuroprotectant for dopaminergic neurons, GDNF is proven to be effective for PD, with clinical trials currently in progress [1]. Although a randomized controlled trial published resulted in negative outcomes, and controversy about the efficacy and safety of the treatment still remains, several pilot studies revealed the validity of continuous intraputaminal GDNF infusion to patients with PD. According to a few clinical trials on transplantation therapy in PD cases, the transplanted potential of implanting microparticles or transfected cells in the human brain is limited by their size, which is substantially larger than the effective pore size of the extracellular spaces of the brain. Consequently, this is likely to restrict their therapeutic potential [1]. In this study, GDNF liposomes were prepared at first; its permeability *in vitro* BBB model and the uptake in brain *in vivo* were then studied. Though the accumulation of GDNF liposomes in brain is low, GDNF-SSL-T realized the aim of targeted delivery to CNS. We will further explore better methods to increase brain delivery of GDNF liposomes *in vivo* by modified prescription.

The particle size is an important factor that affects the liposome endocytosis in the brain capillary cells [23]. In our study, the size of the prepared GDNF-L and GDNF-SSL-T were all below 90 nm, which indicated a favorable condition for brain transport. In additions, the stability of GDNF liposomes in the plasma has been evaluated by centrifuging method in the preliminary experiment. The leakage percent of GDNF liposomes were no more than 8% at 4 h, which showed a good stability for the following permeability studies.

A primary *in vitro* BBB model is a useful technique for the study of CNS drug transport across the BBB. Studies have shown that the BBB model, established by co-culturing of BCECs and ACs of rat, possessed similar morphology, TEER and permeability characterizations of the BBB [24,25]. The TEER values of the coculture of BCECs/ACs in the study were 360 ± 35 Ωcm^2^, which were close to those reported previously [24,26]. Meanwhile, the permeability of HRP was 0.53% on the coculture BCECs/ACs, verified the validity of the *in vitro* BBB model. There was correlation between the TEER and BBB permeability: the TEER increases while permeability decreases. The nonlinear relationship between BBB permeability and TEER was described in a literature [27]. So, TEER was an essential and simple indicator to estimate the formation of the *in vitro* BBB model. After the BBB model was formed, they were employed to evaluate the permeability of GDNF liposomes. As illustrated in Figure 1, the order of permeability on the *in vitro* model of BBB was GDNF-SSL-T > GDNF-L > GDNF. The results indicated that the permeability of GDNF was increased after it was incorporated into liposomes and could be increased significantly after it was loaded by SSL-T.

To investigate the brain delivery of GDNF, GDNF-L and GDNF-SSL-T, *in vivo* uptake was studied in mouse after intravenous injection at a dosage of 10 μg/100 g. As showed in Figures 2 and 3, the concentrations of GDNF in the serum and brain of the GDNF-SSL-T group were significantly higher than those of the GDNF group and GDNF-L group at all time points, respectively (*p* < 0.01). But there was no distinct difference between the GDNF-L group and the GDNF group. The AUC_(0–1 h)_ of brain GDNF-SSL-T was 8.1 times and 6.8 times more than that of free GDNF and GDNF-L, respectively. The favorable brain delivery of GDNF-SSL-T is probably due to its modification with PEGylated polymer and chemical conjugation with RMP-7. The conventional liposomes are easy to be trapped by the Reticuroendothelial systems (RES) in the blood [28]. With PEGylated polymer, however, sterically stabilized liposomes (SSL) can avoid the identification and clearance by RES that its life time in blood was thus prolonged [29,30] Besides, by combining RMP-7 with 1,2-Dioleoyl-*sn*-glycerol-3-phosphor-ethanolamine-*n*-[poly(ethyle-neglycol)]-hydroxy succinamide (DSPE-PEG-NHS), DSPE-PEG-RMP-7 was obtained and incorporated into the liposomes surface to form the active targeted sterically stabilized liposomes (SSL-T). RMP-7 has a longer circulation time in the blood and better selectivity to the B2 receptor on the BBB [31]. The mechanism of RMP-7 promoting the uptake of drugs in the brain has been demonstrated in literatures [27,32]. Experiments have confirmed that this chemically conjugation would make the drug and RMP-7 arrive BBB at the same time. RMP-7 could induce the efflux of Ca^2+^ in cells, causing brain capillary endothelial cells to be shrunk, the TJ would then be “open” temporarily, and the drug is transported across the “open” TJ into the brain immediately [27,32]. The study also showed that this TJ opening was effective and less toxic when RMP-7 was combined with SSL [27].

## 4. Materials and Methods

### 4.1. Materials and Animals

GDNF was purchased from Peprotech Inc. (Rocky Hill, NJ, USA). Soybean phospholipids (SPC) and cholesterol (CHOL) were obtained from Beijing Chemical Agent Co. (Beijing, China). 1,2-Dioleoyl-*sn*-glycerol-3-phosphor-ethanolamine-*n*-[poly(ethyle-neglycol)]-hydroxy succinamide (DSPE-PEG-NHS) was from Nanocs Inc. (New York, NY, USA). RMP-7 was provided by Alkermes Inc. (Cambridge, MA, USA), and fluorescein isothiocyanate (FITC) was provided by Amresco (Solon, OH, USA). Horseradish peroxidase (HRP) was from Baobanbio Co. (Shanghai, China). Glial cell line-derived neurotrophic factor sandwich ELISA kit (GDNF ELISA kit) was obtained from RayBiotech Inc. (Norcross, GA, USA). Adult male Sprague-Dawley rats were provided by Sun Yat-sen University’s Animal Experiment Center (Guangzhou, China).

### 4.2. Preparation and Properties of GDNF Liposomes

#### 4.2.1. FITC-Labeled GDNF (F-GDNF)

FITC was dissolved in 0.5 mol/L Na_2_CO_3_ buffers (pH 9.5), the FITC solution reacted with GDNF at 4 °C for 6 h in molar ratio 50:1. After that, FITC-labeled GDNF (F-GDNF) was separated from FITC solution on a Sephadex G-25 column (Santa Clara, CA, USA).

#### 4.2.2. Preparation of GDNF Conventional Liposomes (GDNF-L)

Liposomes were prepared by the modified reverse phase evaporation method [27,33]. First, SPC and CHOL (2:1, mol/mol) were dissolved in alcohol and dichloromethane (2:1, *v*/*v*) in a round-bottom flask. The flask was connected to a rotary evaporator(R-201; Shanghai Science and Education Equipment Co., Ltd., Shanghai, China). The organic solvents were evaporated under reduced pressure at 45 °C until a thin lipid film was formed on the inner wall of the flask. The lipid film was hydrated with a buffer salt solution containing HEPES at 45 °C. The hydrating solutions contained 10 μg of GDNF and 1.5 g of sucrose. After vortexing, the liposomes were passed through a high-pressure homogenizer at a pressure of 1000 bars for 10 cycles, and finally, the GDNF-L was finally obtained.

#### 4.2.3. Preparation of GDNF Targeted Sterically Stabilized Liposomes (GDNF-SSL-T)

First, RMP-7 reacted with DSPE-PEG-NHS at a molar ration of 1:6 as described previously [27,34], and the reaction product was checked by Matrix-Assisted Laser Desorption Ionization Time of Flight Mass Spectrometry. The result demonstrated that RMP-7 was transformed into DSPE-PEG-RMP-7, that DSPE-PEG-NHS had combined with RMP-7 at a molar ration of 1:1, and superfluous DSPE-PEG-NHS would be hydrolyzed to be DSPE-PEG. Then the mixture of DSPE-PEG and DSPE-PEG-RMP-7 was incorporated into the membrane of GDNF-L by over-night incubation at 4 °C with gentle stirring to form GDNF-SSL-T.

#### 4.2.4. Properties of GDNF Liposomes

GDNF liposomes were separated from un-encapsulated GDNF by the Sepharose CL-4B column. GDNF was labeled with FITC, then the concentration of GDNF was determined by the spectrofluorometer. The encapsulation efficiency (ee%) and recovery efficiency (re%) of GDNF liposomes was calculated. The size of liposomes was examined by laser light scattering spectroscopy (ALV/DLS/SLS-5022F, Langen, Germany).

### 4.3. Establishment and Evaluation of the *in Vitro* BBB Model

#### 4.3.1. Primary Culture of Rat Brain Capillary Endothelial Cells (BCECs) and Astrocytes (ACs)

The BCECs from the brain cortices of SD rats were isolated by a combination steps of mechanical disintegration, enzymatic digestion, and centrifugation, and were cultured as described previously [24,35]. Briefly, the BCECs were cultured in complete culture medium containing 20% FBS, Gluta MAX-1 (4 mmol/L), HEPES (20 mmol/L), Heparin (40 U/mL), Pen-Stre (100 U/mL–100 μg/mL), ECGS (100 μg/mL) and bFGF (10 ng/mL), and seeded onto 1% gelatin-coated culture flask. The BCECs were characterized by immunocytochemistry and morphology under Inverted Phase Contrast Microscope.

Primary cultures of ACs were initiated from rat brains, as described previously [36]. The ACs were culture in medium containing 15% FBS, Gluta MAX-1 (4 mmol/L) and Pen-Stre (100 U/mL–100 μg/mL), and seeded on flask.

#### 4.3.2. The Development of an *in Vitro* Model of the BBB

BCECs and ACs were cocultured in a “contact through feet” model. In a 12-well cell culture insert with 1 μm-diameter microporous polyethylene terephthalate (PET) membrane (Falcon, 1 μm pore size, 10.5 mm diameter, 0.9 cm^2^ surface area), astrocytes were transferred at second passage on the bottom side at a density of 5.0 × 10^5^ cells/mL by placing theinsert upside down. After the astrocytes attached firmly 4 h later, and then the membrane was turned over and placed in 12-well culture plate. The complete medium for ACs culture containing 15% FBS was added and changed every other day. Five days later, BCECs at the second passage were seeded on the upper side of the inserts at a density of 2.5 × 10^5^ cells/mL. Complete medium for BCEC culture containing 20% FBS was placed on and changed every other day [24]. After 5–7 days coculture, the tightness of the monolayer was assessed by measuring the transendothelial electrical resistance (TEER) using a TEER instrument (Word Precision Instruments, Inc., Sarasota, FL, USA) and viewed with scan electron microscope (SEM, JSM-5600 LV, JEOL, Tokyo, Japan) at instrumental magnification of 10,000 folds. HRP (RZ = 3.0–3.5, enzyme activity > 265 U/mg, *M*_w_ = 44 kDa) was selected as an indicator to evaluate the permeability of the BBB model, as reported by Xie *et al*. [27].

### 4.4. The Permeability of GDNF Liposomes on the BBB Model

To study the influence of different liposomes on GDNF permeability of the BBB model, three groups were set: GDNF, GDNF-L and GDNF-SSL-T. GDNF was labeled with FITC to facilitate the measurement of content by a spectrometer. In each group, GDNF content was 1.0 μg in the donor chamber. GDNF, GDNF-L or GDNF-SSL-T were dissolved into the experimental culture medium (complete endothelial cell culture medium without ECGS), separately. The primary complete medium in the donor chamber was substituted by 500 μL experimental culture medium, and another 1500 μL experimental culture medium was added into the acceptor chamber so that liquid on both sides of the cell insert was at the same level, which can avoid hydrostatic pressure. Samples of 100 μL were taken from the acceptor chamber at 0.5, 1.0, 2.0, 4.0, 8.0 h and replaced by 100 μL of fresh experimental culture medium each time. Cells were kept under culture conditions (37 °C, 5% CO_2_, and saturated humidified atmosphere) during the whole transporting experiment. In the group of GDNF-L and GDNF-SSL-T, the samples taken from the acceptor chamber were treated by Triton X-100 to release F-GDNF from the liposomes. The concentration of GDNF was measured by the spectrofluorometer as described above. The permeability percent (*P*%) of GDNF liposomes on the *in vitro* model was calculated by formula, has been described in the references [27].

### 4.5. The Transporting Ability of GDNF Liposomes across the BBB *in Vivo*

The *in vivo* studies were approved by Sun Yat-sen University Animal Ethical Experimentation Committee according to the requirement of the National Act on the Use of Experimental Animals (Beijing, China).

Fifty-four adult SD rats (250 ± 10 g) were randomly divided into three groups and administrated GDNF, GDNF-L, GDNF-SSL-T, respectively. The drug was administrated through tail vein at a dose of 10 μg GDNF per 100 g body weight in each group. After injection, the blood was collected at a given intervals of 0.25, 0.5, 1.0 h after injection. The rats were then sacrificed and their brains were obtained. Washed by cold still water, these brain tissues were then weighted and stored at −20 °C before measurement of GDNF concentrations as described later.

The concentrations of GDNF in the blood samples and brain tissue were measured by a GDNF ELISA kit. First, blood samples was centrifuged at 3000× *g* for 10 min to obtain serum, and treated with 0.3% Triton X-100 before measurement. While the brain tissues samples (50 μg) were resuspended (1:10, *w*/*v*) in a buffer consisting of a balanced salt solution of Dulbecco/Tris (pH 7.4) and 0.2% Triton X-100 and homogenized at 4 °C (Ultrasonic Processor, 750 Watt Model, St. Louis, MO, USA) for 40 s per sample. All the samples were centrifuged at 14,000 g for 10 min before collecting and concentrating the liquor supernatant [37]. After that, GDNF ELISA kit was used to measure GDNF content according to the manufacturer’s instruction.

### 4.6. Statistical Analysis

Data were expressed as mean ± standard deviation (SD). Student’s *t*-test was used for group comparisons. Values of *p* < 0.05 were considered significant.

## 5. Conclusions

GDNF-L and GDNF-SSL-T were prepared by modified reverse phase evaporation method, and all their sizes were below 90 nm. BBB model was established and evaluated by TEER and permeability to study the permeability of GDNF liposomes *in vitro*. The results *in vitro* showed that the liposomes, especially SSL-T, could facilitate GDNF transportation across BBB. The distribution of GDNF *in vivo* was also studied. Free GDNF and GDNF-L were eliminated rapidly in the circulation. While GDNF-SSL-T, which modified with PEGylated polymer and chemically conjugated with RMP-7, has a longer circulation time in the blood and favorable brain delivery. We can conclude that GDNF-SSL-T realized the aim of targeted delivery of therapeutic proteins to CNS.

## Figures and Tables

**Table 1.** Characterization of GDNF Liposomes (*n* = 6).

	Mean size (nm)	Encapsulation efficiency (%)	Recovery efficiency (%)
GDNF-L	85.65 ± 0.75	33.25 ± 0.92	99.15 ± 5.81
GDNF-SSL-T	81.50 ± 0.66	37.46 ± 1.75	97.38 ± 4.09
